# External sinus lift with simultaneous implant placement for severely atrophic maxilla: optimization of staged surgical approaches

**DOI:** 10.1038/s41405-025-00380-2

**Published:** 2025-11-29

**Authors:** Hanqing Liu, Hansen Liu, Qingkun Jiang

**Affiliations:** 1 Jiangxi Provincial Key Laboratory of Oral Diseases, Department of Stomatology, The First AffiliatedHospital, Jiangxi Medical College, Nanchang University, Nanchang, Jiangxi China; 2 Hangzhou Qiantang Dental Hospital, Hangzhou, Zhejiang China

**Keywords:** Peri-implantitis, Oral diseases

## Abstract

**Background:**

In previous studies, immediate implant placement in molar regions has been widely applied.

**Purpose:**

To study the clinical effect and feasibility of simultaneous implantation of implants combined with lateral maxillary sinus floor elevation in the maxillary molar region with severe bone defects.

**Materials and methods:**

Patients requiring lateral maxillary sinus elevation (LMSE) surgery in the maxillary molar region were selected. The patients (residual bone height, RBH < 3 mm) were randomly divided into two groups: the experimental group underwent simultaneous implant placement combined with LMSE; the control group first received bone augmentation via LMSE, followed by delayed implant placement. The outcome indicators included implant success rate, surgical complications, and measurements of Cone Beam Computer Tomography (CBCT) data.

**Results:**

The implant survival rate of both groups was 100%. One patient in each group experienced a maxillary sinus membrane perforation. From T1 (immediately after surgery) to T2 (9 months after surgery), both the bone height and bone width decreased in both the experimental group and the control group, and the bone resorption at the three sites (IBSH, MBH, DBH) in terms of bone height was greater than that in terms of bone width. The minimum bone resorption was observed at the IBSH site in both group. The amount of bone resorption in the experimental group at the above three sites was less than that in the control group, with statistical differences(*p* < 0.05). There was no statistical difference in the amount of bone width resorption between the two groups(*p* > 0.05).

**Conclusion:**

In cases where the bone volume is less than 3 mm, the method of simultaneous implantation with LMSE is feasible.

## Introduction

The loss of maxillary posterior teeth often results in insufficient residual bone volume due to factors such as alveolar bone resorption caused by periodontal or periapical disease, as well as pneumatization of the maxillary sinus. For patients with a residual bone height of the maxillary sinus measuring ≥6 mm, a kit of tapered titanium implants with increasing diameters, as proposed by Summers [1]^,^ is commonly used clinically. This involves tapping the sinus floor through the implant hole at the crest of the edentulous alveolar ridge to elevate the maxillary sinus floor. Although this method has the advantages of short surgical operation time and minimal surgical trauma, it is prone to causing headache and discomfort in patients. Moreover, due to poor visibility, it is likely to lead to sinus membrane perforation. Additionally, it is unsuitable for patients with excessively low residual bone height (RBH) or irregular sinus floor morphology.

The current expert consensus indicates that when the RBH is 4–6 mm, simultaneous implantation with lateral window maxillary sinus floor elevation can be adopted; when the RBH is less than 3 mm, delayed implantation should be performed after lateral window maxillary sinus floor elevation first [2]. The lateral window technique for sinus floor elevation was first proposed in the 1970^s^ [3]. Long-term clinical follow-up has demonstrated its high success rate, with 95% at 10 years and 85% at 20 year^s^ [4], and it has since been widely adopted as a classic surgical procedure. However, compared with maxillary sinus floor elevation via the transalveolar approach, this technique also carries risks such as greater surgical trauma, longer operation times, and a higher likelihood of causing patient discomfort and postoperative complications [5].

Maxillary sinus floor elevation typically requires graft materials that possess osteoconductivity and osteoinductivity. Autologous bone grafts, bone substitutes, or a combination of both can be utilized as intrasinusal filling materials for maxillary sinus elevation [6, 7]. Autologous bone is recognized as the gold standard for bone augmentation [8, 9]. However, harvesting autologous bone necessitates additional surgical incisions. When the demand for autologous bone is low, it can be obtained from the maxilla (maxillary tuberosity) or mandible (retromolar area or chin). When a larger amount is required, additional autologous bone must be harvested from the ilium or skull, resulting in a greater postoperative trauma response in patients [10, 11]. Therefore, during clinical surgeries, bone substitutes are more frequently used as the preferred graft materials.

Bone substitutes are categorized into allogeneic, xenogeneic, and synthetic bone materials. Xenogeneic bone graft materials and synthetic bone materials act solely as scaffolds for new bone growth, possessing only osteoconductive properties. Over time, the bone graft materials will be partially replaced by active bone tissue. Demineralized, freeze-dried allogeneic bone, however, has osteoinductive properties [12]. Xenogeneic bone can be sourced from various materials, such as bovine and porcine bone. These materials undergo processes including decellularization, deantigenization, sterilization, and structural optimization before being applied in clinical practice. Xenogeneic bone offers several advantages, including a wide range of raw material sources, low immunogenicity, and the preservation of the collagen fiber network and inorganic minerals found in natural bone. Nevertheless, it also presents numerous disadvantages, such as residual immunogenicity, the absence of osteoblasts, and potential risks associated with the transmission of pathogens.

In addition to the influence of bone graft materials, the key to bone formation in bone grafting lies in the stability of the grafting site [13]. However, the osteogenic function of the maxillary sinus mucosa following elevation remains unclear. Currently, the academic community generally believes that the bone at the maxillary sinus floor and the lateral wall can provide osteoblasts and growth factors, thereby activating the initial process of new bone formation [14].

Currently, there are limited comparative studies on patients with severe bone loss (less than 3 mm) in the posterior maxillary region, comparing simultaneous implantation with lateral window maxillary sinus floor elevation to the traditional method of first performing an intrasinusal bone graft via lateral window elevation followed by implant placement. This study aims to provide clinical research support by evaluating the therapeutic effects of the two surgical methods.

## Materials and methods

### Patients selection

This study included patients treated at the Implant Department of Hangzhou Qiantang Dental Hospital who required a Local Maxillary Sinus Elevation (LMSE) procedure from August 2021 to May 2024. All participants were thoroughly informed about the surgical procedures and associated risks, and they provided their informed consent by signing the necessary forms.

This study complied with the revised World Medical Association Declaration of Helsinki and was approved by the Ethics Committee of the First Affiliated Hospital of Nanchang University (Approval No. IITS2025481).

**The inclusion criteria for this study were as follows:**

1. Age of 18 years or older;
2. Maxillary molars that have lost their retention value due to trauma, caries, pulp disease, periapical disease, or periodontal disease;
3. More than three months have elapsed since the extraction of maxillary molars;
4. Residual bone height (RBH) of 3 mm or less;
5. Ability to maintain good oral hygiene;
6. Thickness of the lateral wall bone of the maxillary sinus <less than 3 mm.

**The exclusion criteria for this study were as follows:**

1. Maxillary sinus cysts and cystic lesions with clinical symptoms;
2. Current or previous use of bisphosphonate drugs;
3. Pregnant or lactating women;
4. History of radiotherapy for head and neck tumors within the past 5 years;
5. Acute or chronic maxillary sinus inflammation;
6. Requirement for horizontal bone augmentation in addition to LMSE combined with intrasinusal bone grafting;
7. Smoking more than 20 cigarettes per day.

All patients in this study were screened by the surgical operators. A total of 98 patients who met the inclusion criteria were selected from 325. The purpose, significance, procedure, and surgical risks of the study were explained to the patients. Of these, 72 patients consented to participate. The 72 patients were then randomly assigned numbers by another stomatologist, and Windows Random Number Generator Professional Edition 1.91 (Segobit Software, Redmond, Washington, USA) was utilized to allocate 36 patients to the experimental group and 36 to the control group.

A priori power analysis using GPower 3.1 (effect size d = 0.65, α = 0.05, power = 0.80) indicated that 30 patients per group would provide adequate power to detect clinically significant differences. The sample size was determined based on the primary outcome of implant survival rate. While this provides adequate power for the primary comparison, it may limit the robustness of subgroup analyses.

### Surgical procedures

Routine oral specialized examinations; clinical laboratory tests including blood routine, coagulation routine, blood glucose, and screening for infectious diseases (such as hepatitis B, syphilis, and AIDS; performing CBCT and selecting implants with appropriate diameter and length based on the three-dimensional CBCT data of the affected teeth; full-mouth scaling. All surgeries in this study were performed by the same experienced surgeon.

Prophylactic antibiotics were administered 1 h prior to surgery: 2 grams of amoxicillin combined with 0.25 grams of ornidazole; an oral compound chlorhexidine mouthwash was used three times, with each use lasting 30 s. The surgery was conducted under local anesthesia using 4% articaine. Patients were briefed on the treatment plan and signed the informed consent form.

**Fig. 1:**
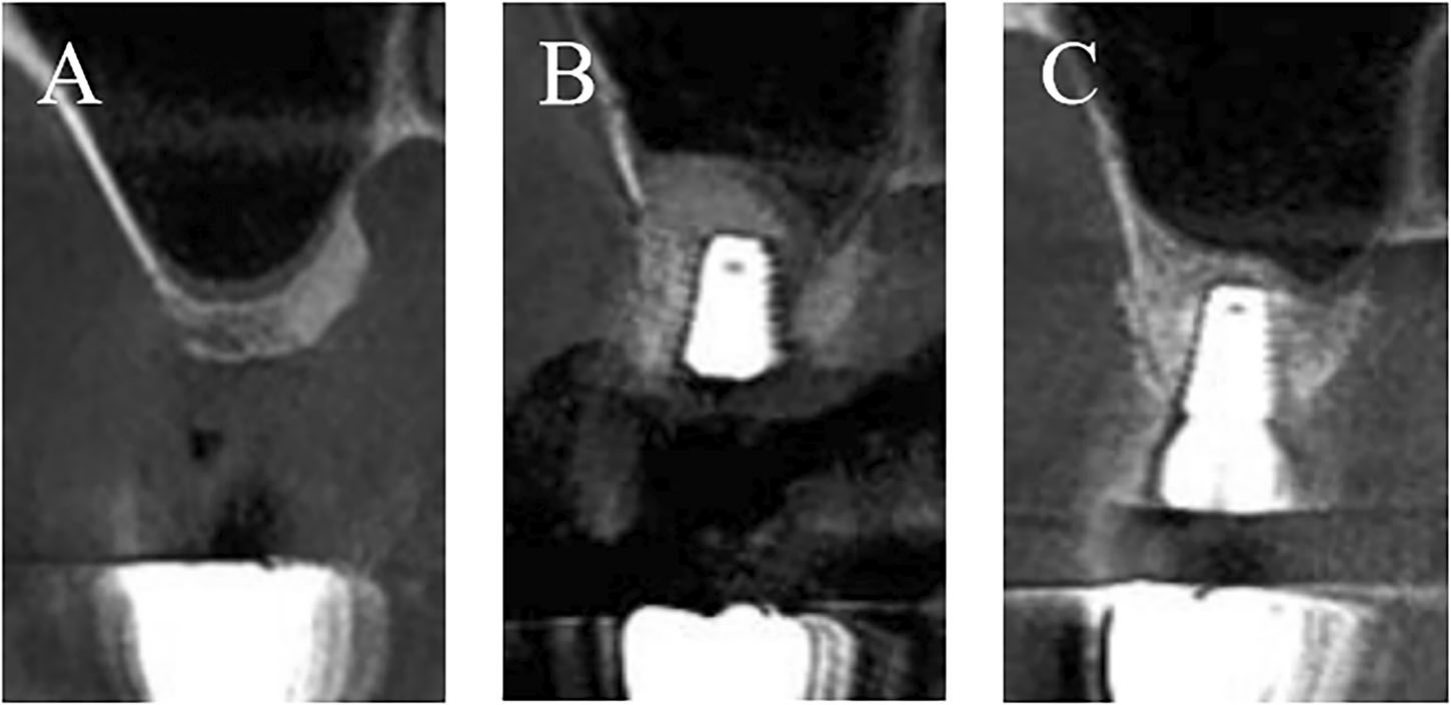
CBCT images of implant placement procedures in the experimental group. **A** Preoperative baseline scan; (**B**) Immediate postoperative assessment; (**C**) Second-stage evaluation.

In the experimental group, an alveolar ridge crest incision and two buccal releasing incisions were made, and a full-thickness mucoperiosteal flap was reflected to expose the buccal bony wall of the maxillary sinus. After using the Dentium external sinus lift kit (Dentium, South Korea) to abrade the buccal wall, the Schneiderian membrane was carefully and evenly elevated. Gelatin sponges were placed into the elevated cavity, and the gelatin sponges were removed after the implant site was reamed and prepared step by step. After visually inspecting and checking the integrity of the Schneiderian membrane via the Valsalva maneuver, deproteinized bovine bone matrix large-particle bone graft (Bio-Oss®, Geistlich AG, Switzerland) was implanted. Then the implant was inserted and its primary stability was measured. A resorbable bovine collagen membrane (Bio-Gide®, Geistlich AG, Switzerland) was used to cover the surface of the buccal bone window. The wound was sutured with 4-0 sutures (Fig.1).

**Fig. 2:**
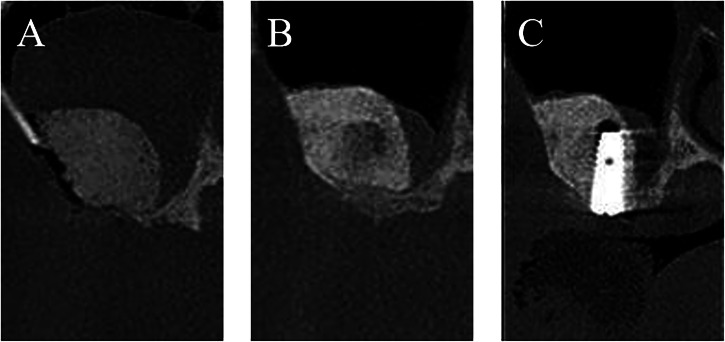
CBCT images of implant placement procedures in the control group. **A** The day of bone augmentation; (**B**) 6 months after bone grafting; (**C**) 3 months after implant placement.

Patients in the control group underwent the same maxillary sinus elevation procedure as the experimental group. The difference was that only a large-particle bone graft was implanted during the initial surgery, without the placement of implants. Six months following the first surgery, implants were inserted, and a secondary surgical treatment was performed three months after the implant placement (Fig. 2).

**Fig. 3:**
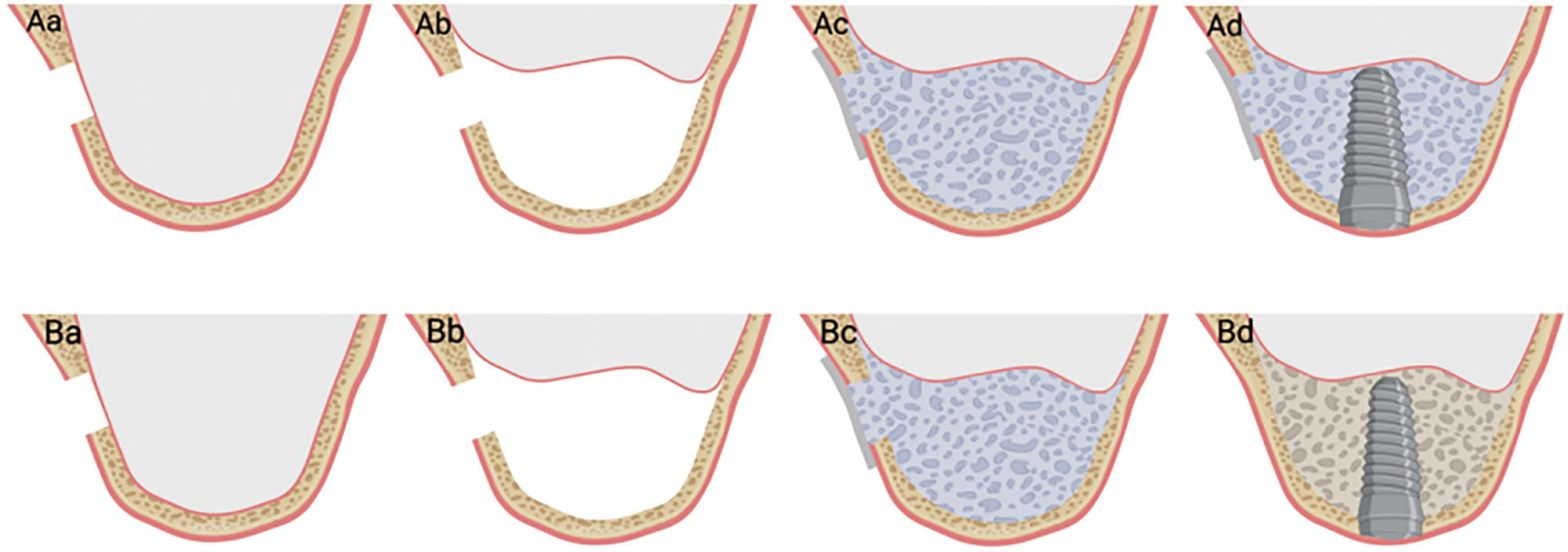
Schematic diagram of the surgical procedure. Experimental group (**Aa**–**Ad**), Control group (**Ba**–**Bd**). **Aa** Grind away the maxillary sinus bone wall; (**Ab**) Dissect the maxillary sinus mucosa; (**Ac**) Fill with bone powder and cover with a collagen membrane; (**Ad**) Simultaneous implant placement; (**Ba**) Grind away the maxillary sinus bone; (**Bb**) Dissect the maxillary sinus mucosa; (**Bc**) Fill with bone powder and cover with a collagen membrane; (**Bd**) Implant placement after 6 months.

The diagram below is a schematic illustration of the surgical procedures for the experimental group and the control group (Fig. 3).

### Postoperative management

All patients were administered cephalosporin and metronidazole antibiotics for a duration of 5 to 7 days. They underwent a follow-up examination 14 days post-surgery for the removal of sutures and to assess wound healing. Should patients experience pain in the surgical area, they were instructed to take 300 mg ibuprofen sustained-release capsules orally, twice daily. Additionally, they were to use 0.2% chlorhexidine for oral rinsing, maintaining the rinse for 1 min, twice a day, for a period of 2 weeks. Patients were advised against engaging in activities such as cheek puffing, blowing, and forceful nose blowing following the surgery.

### Evaluation criteria and methods

#### Implant survival rate

The evaluation of the implant survival rate was based on the success criteria for osseointegration proposed by Buser [15]:

·No displacement of the implant

·No persistent pain, discomfort, or paresthesia

·No peri-implant infection

·No mobility of the implant

#### Surgical complications

The maxillary sinus mucosa was inspected for rupture using visual examination and the Valsalva maneuver. Postoperative symptoms were monitored, including increased nasal discharge, purulent or bloody secretions within the nasal cavity, nasal odor, fever, headache, facial tenderness, and other indications of maxillary sinus infection.

#### CBCT measurement

All patients underwent CBCT at three time points: T0 (before surgery), T1 (immediately after surgery), and T2 (nine months post-surgery). All CBCT scans were conducted by the same radiologist. Each CBCT measurement was taken three times, and the average value was used for analysis. All data measurements were performed by a single individual.

**Fig. 4:**
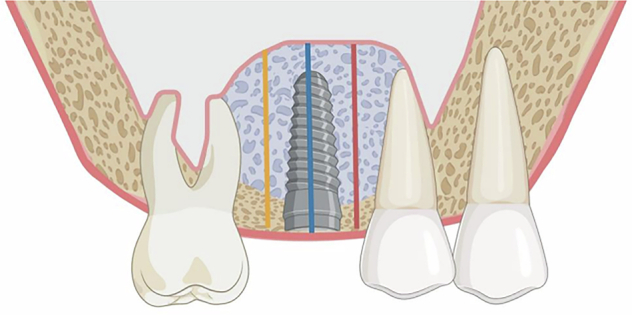
Schematic diagram of bone height measurement at T2 and T3. Blue line (ISBH): Implant Site bone height, which refers to the distance from the implant apex to the most apical position of the graft material along the longitudinal axis of the implant at T0, T1, and T2. Red line (MBH): Mesial bone height, the distance from the lower margin of the alveolar bone at 1 mm away from the mesial margin of the implant to the apex of the bone graft material. Yellow line (DBH): Distal bone height, the distance from the lower margin of the alveolar bone at 1 mm away from the distal margin of the implant to the apex of the bone graft material.

**Fig. 5:**
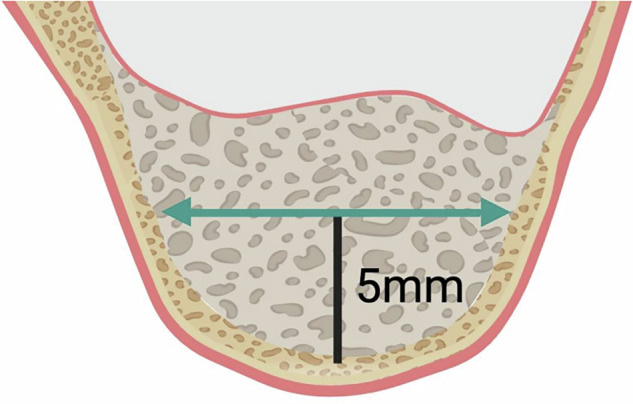
Schematic diagram of bone width measurement at T2 and T3. Green Line (BW): Bone width, referring to the buccolingual width of the bone graft material at a position 5 millimeters away from the maxillary sinus floor along the long axis of the implant.

At T0, the initial bone height was measured in both the test group and the control group. At T1 and T2, both groups were measured for the bone tissue height at the implant long-axis position (ISBH), 1 mm mesial to the implant (MBH), 1 mm distal to the implant (DBH) (Fig. 4), and bone width (BW) (Fig. 5).

### Statistical analysis

The data in this study are expressed as mean ± standard deviation. The primary outcome was the implant survival rate. The secondary outcomes were the measurements of alveolar bone resorption in horizontal and vertical directions at four pre-specified sites (ISBH, MBH, DBH, BW). Since multiple comparisons were performed on these secondary outcomes, a Bonferroni correction was applied to control the family-wise error rate. The adjusted significance level was set at *p* < 0.0125 (0.05/4 comparisons). Independent sample *t*-tests were used to evaluate the changes in bone dimensions between groups at T1 and T2. All statistical analyses were performed using SPSS Version 26 (SPSS; IBM, Chicago, Illinois, USA), and all tests were two-tailed.

### Ethics approval

This study was conducted in accordance with the ethical principles of the revised World Medical Association Declaration of Helsinki. The study protocol was reviewed and approved by the Ethics Committee of the First Affiliated Hospital of Nanchang University (Approval No. IITS2025481). Prior to inclusion in the study, all participants provided written informed consent after being fully informed about the surgical procedures, potential risks, and benefits.

## Results

### Basic characteristics

**Fig. 6:**
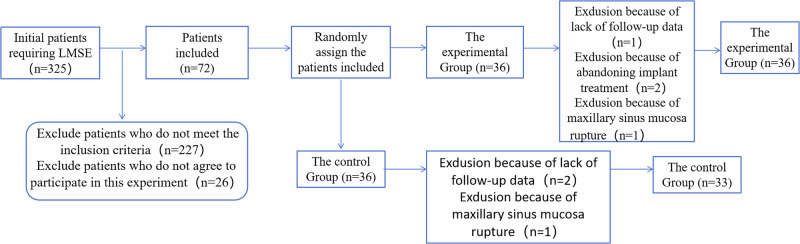
Flow chart for the selection of patients.

A total of seven patients were excluded from the experiment and not included in the statistical analysis: one patient in the experimental group and two patients in the control group were unable to complete subsequent treatment and follow-up due to traveling to other places; two patients in the experimental group gave up implant treatment and switched to fixed bridge repair during the experiment and withdrew from the study; one patient in the experimental group and one patient in the control group withdrew from the experiment due to maxillary sinus mucosa rupture. A total of sixty-five patients (78 implants) completed this study and received subsequent follow-up (Fig. 6). The groups showed comparable baseline characteristics (age, gender, bone volume, etc.; *P* > 0.05), with detailed metrics in Table 1.

**Table 1:** Basic characteristics of patients and implants in the experimental group and the control group.

Variables	Test group (*n* = 32)	Control group (*n* = 33)
Age (years)	42.7 ± 10.8	45.6 ± 12.1
Man/Women (*n*)	17/15	16/17
Maxillary first molar/Maxillary second molar (*n*)	22/10	19/14
Available bone (mm)	2.43 ± 0.57	1.97 ± 0.55
Smoking habits		
Smokers	3	5
Former smokers	13	11
Never smoked	16	17
Implant diameter (mm)		
4.0	12	15
4.5	26	25
Implant length (mm)		
8	18	17
10	20	23

### Implant survival rate and surgical complications

The initial stability of implants after placement in the experimental group ranged from 5 to 40 N·cm, while that in the control group ranged from 25 to 45 N·cm. According to the osseointegration success criteria proposed by Buser, before implant loading in this study, the implant survival rate was 100% .

In this study, a patient in the experimental group experienced a rupture of the maxillary sinus mucosa during surgery, with the rupture measuring between 3 to 5 mm in width. The fenestration area was expanded intraoperatively, the surrounding maxillary sinus mucosa was released, and the damaged maxillary sinus mucosa was covered with an absorbable collagen membrane. Implant placement was carried out following bone grafting. The patient exhibited no nasal bleeding or maxillary sinus inflammation postoperatively, and radiographs taken six months later indicated good healing within the maxillary sinus.

A patient in the control group experienced increased clear nasal mucus one week post-surgery, without any tenderness upon palpation of the lateral wall of the maxillary sinus. CBCT imaging indicated a rupture of the distal mucosa in the bone graft area of the maxillary sinus. The treatment consisted of administering Fuma Nasal Drops (3 drops, three times daily) to the affected nostril for one week. The symptoms improved but did not completely disappear after one week, and they fully resolved approximately one month later. Radiographs taken six months post-surgery showed that although some bone graft material had been lost, the remaining bone volume was still adequate for implant placement, and there was no significant difference in bone density compared to other patients in the control group.

### CBCT measurement

At T0, there was no statistically significant difference in the remaining bone height between the two groups (experimental group: 2.43 ± 0.57 mm, control group: 1.97 ± 0.55 mm) (*p* > 0.05). At T1, the bone heights of the control group at the three sites (ISBH = 12.61 ± 0.31 mm, MBH = 12.18 ± 0.55 mm, DBH = 12.21 ± 0.28 mm) were slightly higher than those of the experimental group (ISBH = 12.51 ± 0.77 mm, MBH = 11.92 ± 0.39 mm, DBH = 11.95 ± 0.47 mm); the bone width of the experimental group (BW = 9.83 ± 0.56 mm) was slightly smaller than that of the control group (BW = 10.31 ± 0.35 mm). At T2, the bone heights of the control group at the three sites were: ISBH = 11.51 ± 0.22 mm, MBH = 10.45 ± 0.18 mm, DBH = 10.46 ± 0.27 mm; the bone heights of the experimental group at the three sites were: ISBH = 12.31 ± 0.58 mm, MBH = 11.49 ± 0.36 mm, DBH = 11.56 ± 0.44 mm; the bone width of the experimental group (BW = 9.70 ± 0.58 mm) was slightly smaller than that of the control group (BW = 10.25 ± 0.36 mm). From T1 to T2, both the bone height and bone width decreased in both the experimental group and the control group, and the bone resorption at the three sites (IBSH, MBH, DBH) in terms of bone height was greater than that in terms of bone width (BW) (Table 2).

**Table 2:** The height and width of alveolar bone at time points T1 and T2 measured by CBCT (mean ± SD).

Time point	Test group				Control group			
	ISBH (mm)	MBH (mm)	DBH (mm)	BW (mm)	ISBH (mm)	MBH (mm)	DBH (mm)	BW (mm)
T1	12.51 ± 0.77	11.92 ± 0.39	11.95 ± 0.47	9.83 ± 0.56	12.61 ± 0.31	12.18 ± 0.55	12.21 ± 0.28	10.31 ± 0.35
T2	12.31 ± 0.58	11.49 ± 0.36	11.56 ± 0.44	9.70 ± 0.58	11.51 ± 0.22	10.45 ± 0.18	10.46 ± 0.27	10.25 ± 0.36

**Fig. 7:**
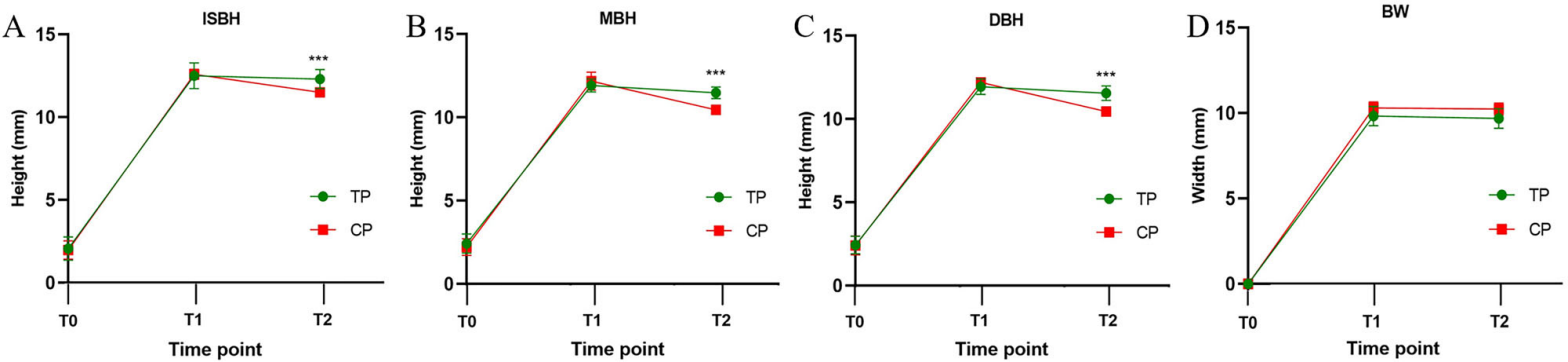
Changes in bone tissue over time in the experimental and control groups. **A** ISBH (Inferior Sinus Bone Height), **B** MBH (Middle Sinus Bone Height), **C** DBH (Distant Sinus Bone Height), and **D** BW (Bone Width). Time points: T0 (before surgery), T1 (immediately after surgery), T2 (9 months after surgery). Green boxes (TP) represent the test group undergoing simultaneous implant placement with LMSE; red boxes (CP) represent the control group receiving bone augmentation via LMSE followed by delayed implant placement. Data are presented as mean ± SD.

In both the experimental group and the control group, bone resorption at the IBSH site was the least, and bone resorption at the MBH and DBH sites was nearly equivalent. When comparing the experimental group with the control group, the bone resorption at the IBSH site was 0.20 ± 0.43 mm in the experimental group and 1.95 ± 0.39 mm in the control group, with a statistically significant difference between the two groups (*p* < 0.01); the bone resorption at the MBH site was 0.43 ± 0.30 mm in the experimental group and 1.72 ± 0.58 mm in the control group, with a statistically significant difference between the two groups (*p* < 0.05); the bone resorption at the DBH site was 0.38 ± 0.32 mm in the experimental group and 1.75 ± 0.37 mm in the control group, with a statistically significant difference between the two groups (*p* < 0.01); the bone resorption of BW was 0.13 ± 0.12 mm in the experimental group and 0.06 ± 0.03 mm in the control group, with no statistically significant difference between the two groups (*p* > 0.01) (Table 3, Fig. 7).

**Table 3:** Changes in the height and width of alveolar bone from time point T1 to T2 measured by CBCT (mean ± SD).

Variables	ISBH(T1)-ISBH(T2)[mm]	MBH(T1)-MBH(T2)[mm]	DBH(T1)-DBH(T2)[mm]	BW(T1)-BW(T2)[mm]
Test group	0.20 ± 0.43	0.43 ± 0.30	0.38 ± 0.32	0.13 ± 0.12
Control group	1.95 ± 0.39	1.72 ± 0.58	1.75 ± 0.37	0.06 ± 0.03
*P*-value	<0.001	<0.001	<0.001	0.89

## Discussion

For patients with residual bone volume less than 3 mm in the posterior maxilla, maxillary sinus floor elevation is typically employed clinically to augment bone volume in the implant area prior to the placement of implants. This study was a prospective randomized controlled clinical trial. For patients with residual bone height less than 3 mm in the maxillary molar area, the experimental group underwent simultaneous maxillary sinus floor elevation and implant placement. Compared with the conventional surgical treatment in the control group, the results indicated that the implant survival rate was 100% in both groups. Imaging comparisons of bone height changes revealed that both groups experienced increases in bone height and width following sinus elevation, but both subsequently decreased, with changes in bone height being more pronounced than those in bone width. The experimental group received implant placement during the initial surgery, which reduced treatment time, the number of surgeries, and patient discomfort, and demonstrated superior advantages in terms of the amount of bone graft material resorption in the later stages.

The favorable bone remodeling outcomes observed, particularly in the simultaneous group, must be interpreted in the context of the grafting material’s properties. Xenografts, derived from different species, have been widely used in maxillary sinus floor elevation as semi-permanent and slowly resorbing bone-conductive grafts [16]. This is supported by a recent Bayesian network meta-analysis which concluded that xenografts are a reliable choice for sinus augmentation, showing favorable outcomes [17]. Further reinforcing this, a systematic review on graft volume changes concluded that while all materials (including allografts such as DBM or FDBA, synthetic biomaterials like β-TCP, and autografts) undergo some resorption at 6 months, combinations involving xenografts (e.g., with allografts) demonstrated the largest net volumetric gain, underscoring their efficacy in space maintenance [18]. The selection of this material for the present study was deliberate, as its proven efficacy and exceptional capacity for long-term space maintenance are paramount for ensuring the stability of the grafting site in cases of severe bone atrophy (RBH < 3 mm)—the primary focus of this investigation. These properties effectively minimize a key variable, allowing for a clearer evaluation of the surgical protocol (simultaneous vs. staged implantation). In line with the objective of achieving optimal space stability, large-particle xenograft bone powder was uniformly used in all cases. While controversy exists regarding the impact of graft particle size on osteogenesis—with some studies suggesting small particles of allografts may form more active bone [19], while others, such as Testori et al., report superior results with larger particles [20]—the choice of large particles in this experimental design was motivated by their demonstrated clinical efficacy in the authors’ previous bone augmentation surgeries and their theoretical advantage for volume stability. Therefore, the use of large-particle xenograft was considered the most appropriate choice to address the research question at hand.

Ensuring the integrity of the Schneiderian membrane is crucial for the success of maxillary sinus external lift surgery [21]. Schneiderian membrane perforation is the most common surgical complication in maxillary sinus floor elevation, with an incidence ranging from 20% to 44% in the lateral window approach [22]. In the experimental group, one patient experienced perforation due to the excessively small distance between the medial and lateral walls of the maxillary sinus, as well as the excessively small angle formed between the medial wall and the floor of the maxillary sinus. During the external sinus lift procedure, the medial mucosa of the maxillary sinus was damaged, leading to the patient’s withdrawal from this experimental study. Currently, there is no optimal method to ensure the safety of mucosal elevation in this area, and the only measure is to perform gentle manipulation. Even if visual inspection and the Valsalva maneuver show no abnormalities in this area, maxillary sinus mucosal rupture may still occur at a later stage. For such patients, the author places a trimmed absorbable membrane in this area after mucosal elevation, followed by the placement of bone graft material. In the surgical cases over the past five years, no loss of bone graft material due to mucosal damage has occurred in the above-mentioned situations.

A limited surgical field is another significant cause of Schneiderian membrane perforation. A larger bone window can offer improved surgical visibility and operational space, particularly when there are bony protrusions at the maxillary sinus floor or chronic inflammation in the maxillary sinus, which reduces the likelihood of maxillary sinus mucosal rupture. Many scholars have attempted to create larger bone windows, typically measuring 10–15 mm in width and 8–10 mm in height [23, 24]. However, some scholars argue that small bone windows can also provide sufficient vision without compromising surgical procedures [25]. Although small bone windows have benefits such as minimal surgical trauma, limited flap reflection, and mild postoperative complications, the author only employs small bone windows under optimal conditions, which include a flat maxillary sinus floor, the absence of cysts or cystic lesions, and no chronic mucosal inflammation in the maxillary sinus.

Surgical technique and instrument selection are also crucial factors influencing the risk of membrane perforation. Piezoelectric surgery has been widely advocated for sinus elevation procedures due to its selective cutting properties for mineralized tissue, which minimizes the risk of soft tissue damage and may thereby reduce the incidence of Schneiderian membrane perforation [26]. In the present study, however, a conventional rotary instrument was employed for all lateral window preparations. This decision was based on the senior surgeon’s extensive experience and proven proficiency with this technique, which has yielded a consistently low perforation rate in their clinical practice. The primary aim of this study was to investigate the timing of implant placement rather than to compare surgical devices. By standardizing the protocol with a well-controlled, traditional technique, we aimed to isolate the variable of interest. Nonetheless, the use of piezoelectric devices represents a valuable technological advancement that may further enhance the safety margin, particularly for less experienced operators or in cases with challenging sinus anatomy, and should be considered a recommended option in future clinical practice and studies.

The main focus of perforation management is to provide stable coverage for the perforated area, allowing it to accommodate the graft materials [27]. The first step in treating mucosal perforation is to relax the surrounding mucosa, reduce tension in the area, and avoid further tearing. The size and location of the perforation should then be evaluated. For small perforations, self-repair may be achieved through blood clot formation or folding of the maxillary sinus membrane. For larger perforations ( > 5 mm), an absorbable membrane should be used to cover the site, serving as a barrier between the sinus cavity and the graft materials. In cases of extensive perforations ( > 10 mm), it is recommended to use a large absorbable membrane that extends to the lateral wall and is fixed with membrane nails or sutures [28]. Absorbable sutures should be avoided when repairing ruptured membranes, especially for large perforations, as suturing often exacerbates the rupture area.

Intraoperative hemorrhage is a common surgical complication. It is mainly caused by the rupture of well-known blood vessels and other tiny blood vessels involved in the surgical area, especially the alveolar antral artery (AAA) [29]. The alveolar antral artery can be completely intraosseous, partially intraosseous, or located under the periosteum of the lateral wall of the maxillary sinus. The diameter of this blood vessel is usually < 1 mm, while anastomotic branches with a diameter > 2 mm are relatively rare [30]. Rupture of an artery with a larger diameter (2.5–3.0 mm) can lead to severe intraoperative hemorrhage [31]. A large amount of bleeding will seriously obscure the surgical field of view, thereby affecting the duration of the surgical operation and the precision of surgical manipulation, and increasing the risks of postoperative swelling and infection. In this study, partially intraosseous blood vessels and those under the maxillary sinus periosteum usually did not rupture during the operation through careful surgical techniques. Even if partial rupture of these blood vessels occurred, the self-constriction of the blood vessels would not cause massive bleeding. However, when intraosseous blood vessels with a diameter >3 mm cannot be avoided during the operation, methods such as electrocoagulation hemostasis need to be used to seal the blood vessels.

In addition to intraoperative hemorrhage, various complications may arise following maxillary sinus external elevation surgery, such as hematoma, edema, acute maxillary sinusitis, postoperative infection, wound dehiscence with exposure of graft material, and postoperative hemorrhage. In both the experimental and control groups, only edema and facial ecchymosis were observed in patients post-surgery, with none of the aforementioned complications occurring. The factors contributing to postoperative complications are often multifactorial. The primary factors for preventing maxillary sinusitis and postoperative infection include the use of aseptic techniques during surgery and maintaining the integrity of the maxillary sinus mucosa. Furthermore, obstruction of the maxillary sinus ostium is also a significant factor that can lead to maxillary sinusitis. Proper hemostasis at intraoperative bleeding sites and postoperative pressure dressing on the surgical area are also important measures to reduce postoperative edema and hemorrhage.

Another important consideration in planning sinus floor elevation is the management of pre-existing maxillary sinus cysts. Cysts and cyst-like lesions in the maxillary sinus can affect the complexity and prognosis of maxillary sinus floor elevation surgery. Asymptomatic cysts and cyst-like lesions are predominantly maxillary sinus pseudocysts and retention cysts. The accumulation of mucus in these cysts within the lamina propria of the maxillary sinus mucoperiosteum often stretches and thins the mucoperiosteum, thereby increasing the risk of perforation during maxillary sinus floor elevation [18, 32, 33]. For patients without clinical symptoms of the maxillary sinus included in this study, there are two treatment options. First, if maxillary sinus floor elevation is performed and the cyst does not interfere with the maxillary sinus opening, maxillary sinus external elevation can be directly conducted without addressing the cyst. Second, if the cyst hinders the drainage of the maxillary sinus opening after maxillary sinus floor elevation, the cyst must be aspirated or removed prior to the elevation procedure. Maxillary sinus cysts or cyst-like lesions are no longer considered absolute contraindications for maxillary sinus floor elevation; however, surgeons should devise appropriate treatment plans based on X-ray films and patients’ clinical symptoms.

Some scholars have attempted to perform maxillary sinus external lift with simultaneous implant placement in the context of residual bone height (RBH) < 3 mm, but this is limited to maxillae with intact double-layer cortical bone, as intact double-layer cortical bone can provide good primary stability [34]. Studies have also demonstrated that simultaneous implant placement in maxillae with RBH < 3 mm can achieve good primary stability and osseointegration effects through measurements of implant stability quotient (ISQ) values and CBCT imaging [35]. However, some scholars question whether severely atrophied maxillae with RBH < 3 mm can provide good primary stability for implants [36]. The authors also believe that when RBH < 3 mm, it is impossible to ensure that every patient will have good primary stability after implant placement. In this study, some patients had primary implant stability <10 N due to maxillary sinus cortical bone loss and residual bone density classified as Type IV bone, but this did not affect implant osseointegration or subsequent loading. In addition, successful implant osseointegration is related to multiple factors, such as general health status, oral hygiene habits, surgeon experience, implant design and surface treatment, and the influence of bone augmentation materials [37].

When performing maxillary sinus floor elevation and simultaneous implantation in patients with RBH < 3 mm, some scholars believe that the success rate (the 10-year cumulative survival rate of osseointegrated implants is only 53.3%) is much lower than the survival rate (92.9%) of implants simultaneously placed in the maxilla with RBH > 3 mm [38]. Some scholars have also put forward similar views, that is, the long-term survival rate of implants is positively correlated with the remaining bone mass [39]. However, another study with a longer follow-up period and a larger number of included cases showed that the 20-year cumulative survival rate of implants in this procedure was 78.8% [4].

This study has several limitations that should be considered when interpreting the results. First, the sample size, although adequate for the primary outcome, remains relatively small and limits meaningful subgroup analyses (e.g., based on bone density or specific anatomic variations). Second, the single-center, single-surgeon design may affect the generalizability of the findings, and the results should be validated in multi-center settings with larger cohorts. Third, the 9-month follow-up period is sufficient for assessing bone remodeling and implant stability before loading but is insufficient to evaluate long-term outcomes, such as graft resorption and implant success over decades. Furthermore, the study lacked a non-grafted control group for ethical and clinical rationale, which would have provided direct insight into the intrinsic osteogenic capacity of the maxillary sinus under these conditions. Future studies with larger sample sizes, longer follow-up periods, and multi-center designs are warranted to confirm these preliminary findings. Furthermore, the absence of a non-grafted control group limits our ability to precisely delineate the extent of bone loss specifically attributable to the resorptive characteristics of the xenograft material versus the baseline resorption resulting from the surgical trauma of the sinus elevation procedure itself.

## Conclusion

Simultaneous implant placement with lateral maxillary sinus elevation is a viable treatment option for severely atrophic maxillae (RBH < 3 mm), offering reduced treatment time and satisfactory bone stability, though long-term outcomes require further investigation.

## Data Availability

All data required for evaluating the conclusions of this study are included in the manuscript.
